# Thoracic Epidural Anesthesia in Cats: A Retrospective Case Series

**DOI:** 10.3390/vetsci12080738

**Published:** 2025-08-07

**Authors:** Elena Lardone, Alessandra Landi, Greta Martinelli, Paolo Franci

**Affiliations:** Department of Veterinary Sciences, University of Turin, 2 Largo Paolo Braccini, 10095 Grugliasco, Italy; alessandra.landi@unito.it (A.L.); greta.martinelli@unito.it (G.M.); paolo.franci@unito.it (P.F.)

**Keywords:** epidural injection, TEA, ropivacaine, morphine, major surgery

## Abstract

Thoracic epidural anesthesia (TEA) is widely used in human medicine to provide effective perioperative analgesia, yet its application in veterinary species—particularly cats—remains underexplored. This retrospective case study describes the use of TEA in nine client-owned cats undergoing major surgeries. All cats received a single epidural injection of 0.2 mL/kg of 0.5% ropivacaine combined with 0.1 mg/kg morphine at the T12–T13 interspace using a 25 G × 25 mm Quincke needle. Intraoperative physiological parameters were continuously monitored, and postoperative analgesia was assessed using a validated pain scale. Only one cat exhibited inadequate analgesic coverage, likely due to TEA failure. Of the nine cats, seven required minimal to no intraoperative rescue analgesia, while five received postoperative opioids on the day following surgery. Hemodynamic stability was observed in most cases, with no significant complications reported. These findings suggest that TEA is a feasible and effective technique for perioperative pain management in cats undergoing major surgery. Further prospective studies are warranted to confirm these initial findings and investigate the safety of the technique in a larger population.

## Simple Summary

Cats are highly sensitive to pain-induced stress, which often is displayed through behavioral changes such as anorexia and aggression. Perioperative pain management is a very critical component for a cat as a surgical patient. Despite its well-documented benefits in human medicine, thoracic epidural anesthesia (TEA) remains unexplored in cats due to anatomical challenges and demands for technical expertise. This retrospective case study aimed to describe the feasibility, safety, and analgesic efficacy of TEA in cats undergoing major surgeries. Nine cats received 0.2 mL/kg of 0.5% ropivacaine combined with 0.1 mg/kg morphine via TEA at the T12–T13 level. Thoracic epidural anesthesia was associated with stable intraoperative cardiovascular parameters and reduced systemic perioperative rescue analgesia in the majority of cases. Only one cat exhibited inadequate analgesic coverage, likely due to technical failure. No significant adverse effects or complications were observed. These findings support the potential of TEA as a viable analgesic strategy in surgical cats, with implications for improving clinical outcomes, enhancing animal welfare, and advancing the standard of care in veterinary anesthesia.

## 1. Introduction

Thoracic epidural anesthesia (TEA) is widely considered the gold standard for perioperative analgesia in people, particularly in the context of cardiac, thoracic, and cranial abdominal surgical procedures [1]. An extensive body of clinical and experimental research has consistently demonstrated the multifactorial efficacy of TEA. Notably, TEA produces a profound central neural blockade that effectively attenuates the neuroendocrine stress response associated with surgical trauma, thereby mitigating the incidence of perioperative complications [2]. Furthermore, the sympathectomy induced by TEA confers protective effects on vital organ systems, including the cardiovascular, pulmonary, and gastrointestinal systems, while also exerting favorable influences on immune modulation and coagulation dynamics [3]. Consequently, TEA is regarded as a critical factor in enhancing positive postoperative outcomes following major surgical interventions [4].

In veterinary medicine, anatomical limitations in dogs and cats pose technical challenges for epidural needle placement at the thoracic level and TEA remains a relatively under-investigated technique, with limited data available in dogs [5,6,7]. Tonge et al. [7] reported successful use of TEA in a dog undergoing thoracotomy and pulmonary lobectomy, achieving favorable anesthetic and analgesic outcomes throughout the perioperative period. Similarly, Lardone and colleagues [6] demonstrated the effectiveness of TEA for intra- and postoperative pain management during major thoracic and cranial abdominal procedures. These findings suggest that TEA may offer substantial benefits in veterinary anesthesia, mirroring its established role in human medicine.

To the authors’ knowledge, there is currently no published evidence evaluating the application of TEA in cats. Feline patients are generally more susceptible to stress than dogs, and postoperative pain is a key factor capable of eliciting dysfunction across multiple organ systems. In addition, pain and stress can significantly influence behavioral patterns during hospitalization, frequently manifesting as anorexia, inhibition, and aggression [8]. These considerations highlight the critical importance of comprehensive and proactive pain management strategies in cats undergoing major surgical procedures.

In light of the routine implementation of TEA in both dogs and cats at our institution, the present study aims to retrospectively describe our clinical experience with its use in a cohort of cats undergoing a variety of major surgical interventions.

## 2. Case Presentation

Cats that underwent major surgical procedures between December 2021 and March 2025 at the Teaching Veterinary Hospital of the University of Turin (Grugliasco, Italy) were included in this study. Written informed consent was obtained from all owners. Cases were consecutively numbered from one to nine. Demographic data and relevant clinical details are summarized in Table 1.

### 2.1. Treatment

Table 1 shows the anesthesia protocol.

Following induction of general anesthesia, endotracheal intubation was achieved using appropriately sized cuffed tubes. The endotracheal tubes were connected to a circular breathing system, delivering a gas mixture of oxygen and air with a fractional inspired oxygen (FiO_2_) of 0.40. Maintenance of anesthesia was achieved in three cats using sevoflurane and in six cats using isoflurane. The end-tidal halogenated anesthetic concentration (FE’AA) was set at the anesthetist’s discretion to maintain the surgical plane of anesthesia.

All animals underwent volume-controlled intermittent positive pressure ventilation (Primus, Draeger, Germany) to maintain normocapnia, targeting end-tidal carbon dioxide (PE’CO_2_) levels between 35 and 45 mmHg (4.6 and 6 kPa).

Cefazolin (Cefazolina 1 g, TEVA, Milan (MI), Italy) was administered intravenously at a dose of 20 mg/kg within 20 min prior to the initial surgical incision. Lactated Ringer’s solution (Ringer lattato, Fresenius Kabi, Isolandella Scala (VR), Italy) was infused at a rate of 5 mL/kg/h throughout the anesthetic period. Upon confirmation of a stable surgical plane of anesthesia, cats were placed in lateral recumbency, and the thoraco-lumbar area of the vertebral column was clipped and aseptically prepared with chlorhexidine (4%) and alcohol (70%).

### 2.2. Monitoring

During the intraoperative period, a multiparametric monitor (Infinity Delta, Draeger, Lubeck, Germany) was employed to continuously assess cardiovascular variables—including invasive and/or non-invasive systolic, diastolic, and mean arterial pressures (SAP, DAP, and MAP), heart rate, and cardiac rhythm—as well as respiratory parameters, including PE’CO_2_, peak inspiratory pressure, respiratory rate, tidal volume, minute ventilation, FiO_2_, and FE’AA. Esophageal temperature was also monitored.

To maintain normothermia (body temperature > 36 °C), an active warming system (Bair Hugger Warmer Model 505, Augustine Biomedical Design, Cape Town, South Africa) was used throughout the perioperative period. All monitored parameters were manually recorded at 5 min intervals for the duration of anesthesia.

### 2.3. Thoracic Epidural Anesthesia

Cats were positioned in lateral recumbency with slight flexion of the vertebral column, and the dorsal aspect of the body was extended beyond the edge of the surgical table. The spinous and articular processes of the T13 vertebra were identified using the index finger and thumb, respectively. A 25 gauge × 25 mm Quincke spinal needle (Becton, Dickinson and Company, Franklin Lakes, NJ, USA) was introduced on the dependent side at the T12–T13 intervertebral space via a paramedian approach with cephalad angulation, as previously described in humans [9], dogs [10], and cats [11]. Upon encountering the lamina, it was slightly withdrawn and redirected in a ventrocranial–medial direction until resistance from the ligamentum flavum was pierced and perceived. A plain solution of ropivacaine (5 mg/mL; Ropivacaina, Galenica Senese, Monteroni d’Arbia (SI), Italy) at a dose of 1 mg/kg, combined with morphine (10 mg/mL; Morfina Cloridrato, Monico, Venice (VE), Italy) at 0.1 mg/kg, was administered slowly.

### 2.4. Intraoperative Analgesia

The anesthetists were free to use fentanyl (Fentadon 0.05 mg/mL, Dechra, Turin (TO), Italy) and ketamine (Ketavet 100 mg/mL, MSD Animal Health, Rahway, NJ, USA) as needed to stabilize the anesthetic plane. The amounts of analgesics administered during the procedure are shown in Table 1.

### 2.5. Recovery and Postoperative Plan

The urinary bladder was voided manually at the end of the surgery. Meloxicam (Metacam 2 mg/mL, Boehringer Ingelheim Animal Health, Milan (MI), Italy) at 0.3 mg/kg subcutaneously was administered when normothermia was confirmed during recovery with the exception of case 2 that did not receive any anti-inflammatory drugs.

Methadone (Semfortan 10 mg/mL, Dechra, Northwich, UK) was given intramuscularly (0.2–0.4 mg/kg, IM) during the postoperative period as rescue analgesia using the Glasgow Composite Measure Pain Scale short form (intervention level of 5/20 and above) [12]. In the majority of cases, time-point assessments of analgesia were conducted at 1, 3, and 5 h (h) following extubation. Five cats received methadone the day after the surgery and a patient was administered 9 h after the end of anesthesia. Case 3 received methadone at recovery from anesthesia. Postoperative data were incomplete for two cats.

The assessment of motor function was predominantly performed at 1, 3, and 5 h after extubation, using a previously published scoring system [13]. At 3 h and 5 h after extubation, the 5/7 cats and the 2/7 cats respectively showed motor deficit (grade 2 and 3). Complete motor function recovery occurred in all cats within 24 h of surgery.

## 3. Discussion

To the authors’ knowledge, this is the first report to describe the clinical application of TEA in cats.

In this case series, the use of TEA along with general anesthesia contributed to reducing the perioperative use of rescue analgesia in some cats. It is noteworthy that seven cats out of nine did not need intraoperative rescue analgesia or received just a fentanyl dose. Given that several studies demonstrated a correlation between reduced intraoperative rescue analgesia and successful regional nerve blockade [14,15,16], TEA might have provided effective nociceptive control in the present case series.

No reliable conclusions can be drawn regarding the duration or extent of analgesia provided by TEA during the postoperative period. Nonetheless, five cats received methadone the day after surgery and one cat was administered rescue analgesia 9 h after anesthesia. Considering the invasiveness of the procedures performed, this finding may suggest satisfactory analgesic coverage of the single shot TEA in the first 24 h postoperatively.

The epidural injection of local anesthetics results in the blockade of the paravertebral sympathetic chain. While this can occasionally lead to hypotension due to decreased peripheral vasoconstriction, it may also attenuate the sympathetic response to surgical manipulation, thereby enhancing hemodynamic stability [17,18,19]. The hemodynamic stability and minimal intraoperative rescue analgesia (Table 1), which characterized the majority of the cases, might be attributable to TEA.

Case 3 likely represented a failure of the technique (i.e., incorrect needle positioning or suboptimal anesthetic spread), as it was characterized by hemodynamic instability, insufficient intraoperative antinociceptive effect, and inadequate postoperative analgesic coverage. In this case series, the detection of the epidural space exclusively relied upon the loss of resistance (LOR) at piercing the yellow ligamentum. In this perspective, a Tuohy needle offers the characteristic “pop sensation” and allows the use of air or saline for the LOR technique, which helps confirm the epidural space. Due to its very small diameter, the 25 G Quincke needle made it more difficult to rely upon the LOR technique or other methods for verifying the needle position such as the observation of epidural pressure waves.

In this study, the Quincke spinal needle was preferred over the more commonly used Tuohy needle for epidural anesthesia. The 2.5 cm × 25 G Quincke needle is more suitable for TEA in cats, as it requires minimal needle advancement (about 1 cm [11]). On the other hand, the pediatric 5 cm × 22 G Tuohy needle available would be too long (8 cm long, including the hub). The authors therefore consider the Quincke needle safer than the Tuohy needle due to its shorter length and its greater stability after positioning. However, its smaller gauge makes detecting cerebrospinal fluid reflux more challenging, which can be mitigated by using the dependent paramedian approach. The technique requires more skill, but once mastered, it can be performed quickly and safely. While epidural anesthesia in cats offers notable potential benefits, it also carries a risk of spinal cord injury due to the relatively caudal start of the cauda equina, which provides less space for safe needle placement compared to other species [20]. Given the limited number of cases in this study, it was not possible to assess the risks associated with the technique. Additionally, the inability to use the loss-of-resistance method and the Tuohy needle may further increase the likelihood of spinal cord puncture. For these reasons, TEA should be reserved for specialists with appropriate practical training, a thorough understanding of neuraxial techniques, and the ability to manage patients perioperatively to optimize outcomes and minimize complications.

Although the benefits of continuous epidural analgesia are well recognized—such as the ability to administer analgesic drugs intermittently or continuously over several days, and to do so in lower doses due to the proximity of the catheter tip to the target region—it is not without risks. These include potential catheter dislodgement, local inflammation, and contamination of the insertion site [21,22]. The short execution time, easier postoperative management, and lower cost make the single-injection (“one-shot”) technique a valid alternative for cats undergoing major surgeries.

All the cats received 0.2 mL/kg of 0.5% ropivacaine combined with 0.1 mg/kg of morphine. With this volume, anesthesia remained stable in the majority of cases evaluated, and no notable side effects were observed. The efficacy of the volume was demonstrated even in very small cats (case 2 weighted 1 kg). The current study referenced previous research on lumbosacral and thoracic epidural anesthesia in dogs, particularly focusing on the administration of ropivacaine and levobupivacaine combined with morphine. Epidural injection volumes commonly range from 0.1 to 0.22 mL/kg [23]. In a study by Lardone et al. [6], 0.2 mL/kg of 0.5% ropivacaine with morphine resulted in TEA without significant adverse effects, although prolonged sedation was reported in one case. This aligns with findings from Tonge et al. [7], where TEA with 0.5% levobupivacaine and morphine provided effective intraoperative analgesia without the need for additional systemic pain relief. In refrigerated cats, 0.2 mL/kg of diluted contrast agent resulted in an adequate longitudinal spread for effectively covering the thoracic and cranial lumbar spine [11].

The retrospective nature of this study inherently presented certain limitations, including incomplete data, variability in operators, and lack of standardization in perioperative management. The relatively low frequency of major thoracic surgeries in cats further contributes to the scarcity of studies on thoracic epidural anesthesia [23].

## 4. Conclusions

This case series demonstrated that TEA was feasible in cats. At the administered volume (0.2 mL/kg), TEA provided not only effective intraoperative analgesia but also good hemodynamic stability in cats undergoing major surgery.

Future prospective clinical studies involving larger populations and greater standardization of procedures are needed to further evaluate the intraoperative efficacy, potential side effects, and to characterize the extent and duration of postoperative analgesia provided by TEA in cats.

## Figures and Tables

**Table 1:** Patient and medical details, anesthetic protocol, and intraoperative and postoperative information. DSH: domestic short hair; M: male; F: female; n: neutered; y: years; m: months; min: minutes; IM: intramuscular; IV: intravenous; dex: dexmedetomidine; CRI: constant rate infusion; RA: rescue analgesia; h: hours.

Case	Signalment	Medical State	Surgical Procedure and Anesthesia Time	Premedication	Anesthesia Protocol	Intraoperative RA	Intraoperative Events	First Postoperative RA
**Case 1**	DSH Mn 7 y 4 kg	Chylothorax	Thoracotomy at the 10th intercostal space and pericardiectomy 200 min	None	Fentanyl (6 μg/kg IV) + Propofol (8 mg/kg IV) Isoflurane Cisatracurium (0.2 mg/kg IV at the beginning of the surgery)	Fentanyl (1 bolus at 2 μg/kg IV)	None	Methadone (0.2 mg/kg IM) 9 h after the end of surgery
**Case 2**	DSH M 3 m 1 kg	Diaphragmatic hernia Polypnea Weakness	Diaphragmatic herniorrhaphy 50 min	None	Propofol (5 mg/kg IV) + Cisatracurium (0.3 mg/kg IV, as co-induction agent) Sevoflurane	None	None	Methadone (0.4 mg/kg IM) the day after surgery
**Case 3**	DSH Fn 5 y 3.4 kg	Thymoma	Sternotomy 240 min	Alfaxalone (1 mg/kg IM) Methadone (0.3 mg/kg IM) Dex (4 μg/kg IM)	Alfaxalone (2 mg/kg IV) Sevoflurane	Fentanyl (2 boluses at 2 μg/kg IV + CRI at 5 μg/kg/h IV) Ketamine (1 bolus at 0.5 mg/kg IV + CRI at 20 μg/kg/h IV)	Tachycardia Hypertension Hypotension Noradrenaline	Methadone (0.2 mg/kg IM) at recovery from anesthesia
**Case 4**	DSH Mn 1 y 4.6 kg	Diaphragmatic hernia Polypnea	Diaphragmatic herniorrhaphy 135 min	Methadone (0.1 mg/kg IV)	Dex (2 μg/kg IV) + Alfaxalone (1 mg/kg IV) Isoflurane	None	None	Methadone (0.2 mg/kg IM) the day after surgery
**Case 5**	DSH Fn 1 y 2 kg	Fourth aortic duct persistency	Left lateral thoracotomy at the fourth intercostal space 180 min	Methadone (0.1 mg/kg IV)	Dex (2 μg/kg IV) + Propofol (2.5 mg/kg IV) Sevoflurane	Fentanyl (1 bolus at 2 μg/kg IV)	Tachycardia Hypertension Hypotension Bradycardia Atropine	Lack of data
**Case 6**	DSH Fn 5 y 3.8 kg	Chylothorax	Left lateral thoracotomy at the fourth intercostal space 210 min	Alfaxalone (1 mg/kg IM) Methadone (0.3 mg/kg IM) Dex (2 μg/kg IM)	Propofol 2 mg/kg Isoflurane Cisatracurium (0.2 mg/kg at the beginning of the surgery)	Fentanyl (1 bolus at 2 μg/kg IV) Ketamine (1 bolus at 0.5 mg/kg IV)	Tachycardia Hypertension	Lack of data
**Case 7**	DSH Fn 10 y 4 kg	Hyperthyroidism Chronic renal failure Aggressive Behavior	Left-side radical mastectomy 70 min	Alfaxalone (1.5 mg/kg IM) Methadone (0.3 mg/kg IM) Midazolam (0.25 mg/kg IM)	Propofol (2 mg/kg IV) Isoflurane	None	None	Methadone (0.2 mg/kg IM) the day after surgery
**Case 8**	British cat Mn 8 m 4 kg	Peritoneo-pericardial hernia	Herniorrhaphy 120 min	Alfaxalone (1 mg/kg IM) Methadone (0.15 mg/kg IM)	Propofol (3.5 mg/kg IV) Isoflurane	None	None	Methadone (0.2 mg/kg IM) the day after surgery
**Case 9**	DSH Mn 9 y 5 kg	Thymoma	Sternotomy 240 min	Alfaxalone (1 mg/kg IM) Methadone (0.2 mg/kg IM) Dex (3 μg/kg IM)	Propofol (1 mg/kg IV) Isoflurane	None	None	Methadone (0.2 mg/kg IM) the day after surgery

## Data Availability

The data presented in this study are available on request from the corresponding author due to privacy reasons.
